# Adjunctive aerosolized colistin for multi-drug resistant gram-negative pneumonia in the critically ill: a retrospective study

**DOI:** 10.1186/1471-2253-13-45

**Published:** 2013-11-25

**Authors:** Neha M Doshi, Charles H Cook, Kari L Mount, Stanislaw P Stawicki, Erin N Frazee, Heather A Personett, Garrett E Schramm, Heather M Arnold, Claire V Murphy

**Affiliations:** 1 Department of Pharmacy, St. Luke's University Health Network, Bethlehem, PA, USA; 2 Department of Surgery, The Ohio State University Wexner Medical Center, Columbus, OH, USA; 3 Department of Pharmacy, The Ohio State University Wexner Medical Center, Columbus, OH, USA; 4 Pharmacy Services, Mayo Clinic, Rochester, MN, USA; 5 Department of Pharmacy, Barnes-Jewish Hospital, St. Louis, MO, USA

**Keywords:** Colistin, Multiple drug resistance, Pneumonia, Critical illness, *Pseudomonas*, *Acinetobacter*, Aerosolized

## Abstract

**Background:**

The incidence of multi-drug resistant (MDR) gram-negative (GN) organisms including *Pseudomonas* and *Acinetobacter spp* has increased in the last decade, prompting re-evaluation of colistin for the management of these infections. Aerosolized colistin as an adjunct to intravenous therapy is a current option for the management of MDR-GN pneumonia, although data supporting this practice is limited. This study evaluates the efficacy of adjunctive aerosolized colistin in combination with intravenous colistin in critically ill patients with MDR-GN pneumonia.

**Methods:**

A retrospective multi-center cohort analysis comparing critically ill patients with MDR-GN pneumonia who received intravenous colistin (IV) alone or in combination with adjunctive aerosolized colistin (IV/AER) with a primary endpoint of clinical cure at the end of colistin therapy. Secondary endpoints included microbiologic cure, duration of mechanical ventilation, length of stay, and hospital mortality. A post-hoc subgroup analysis was performed for patients with high quality cultures used for diagnosis of MDR-GN pneumonia. Dichotomous data were compared using Fisher’s exact test while the student’s t-test or Mann–Whitney U test were used for continuous variables.

**Results:**

Ninety-five patients met criteria for evaluation with 51 patients receiving IV and 44 receiving IV/AER. Baseline characteristics were similar between the two groups. Twenty patients (39.2%) receiving IV and 24 (54.5%) receiving IV/AER achieved clinical cure (p = 0.135). There was no difference in microbiologic cure rates between the IV and IV/AER colistin groups (40.7vs. 44.4%, p = 0.805). The IV group demonstrated a trend towards higher pneumonia attributable mortality (70.4 vs. 40%, p = 0.055). In the subgroup analysis of patients with high quality respiratory cultures, there was a significantly lower clinical cure rate for those in the IV group as compared to the IV/AER group (31.3 vs. 57.1%, p = 0.033).

**Conclusions:**

Addition of aerosolized colistin to IV colistin may improve clinical cure and mortality for patients with MDR-GN pneumonia. Larger, prospective trials are warranted to confirm the benefit of adjunctive aerosolized colistin in critically ill patients with MDR-GN pneumonia.

## Background

Multi-drug resistant (MDR) gram-negative (GN) organisms such as *Acinetobacter baumannii, Pseudomonas aeruginosa, and Klebsiella pneumoniae* are frequently associated with nosocomial pneumonia in the intensive care unit (ICU). Nosocomial pneumonia caused by these organisms has been associated with increased morbidity and mortality [1]. Mortality for nosocomial pneumonia ranges between 38% and >70%, with even higher rates for MDR-GN organisms [2]. Unfortunately, the incidence of these MDR pathogens has continued to rise over the last several decades [3]. The increased incidence of MDR-GN pathogens and the lack of new effective antimicrobials has contributed to renewed enthusiasm for employing colistin, a polymyxin antibiotic effective against MDR-GN pathogens, as an alternative therapy. In addition, using intravenous colistin as salvage therapy has increased due to reports of clinical efficacy in patients with MDR infections [4]. Colistin is a cationic detergent that damages bacterial cytoplasmic membranes causing leakage of intracellular contents and cell death [5]. This unique mechanism of action makes colistin effective and less susceptible to bacterial resistance mechanisms. Due to significant toxicity of intravenous colistin and concern for inadequate penetration into the lung parenchyma, adjunctive aerosolized colistin is often used for MDR-GN pneumonia [4,6]. Animal models have failed to detect colistin in lung tissue after intravenous infusion, whereas high lung tissue and low systemic concentrations were observed following a single dose of aerosolized colistin [7]. The use of aerosolized colistin has previously been studied in cystic fibrosis (CF) patients, but has only recently been investigated for non-CF nosocomial pneumonia. Currently, there are conflicting data regarding aerosolized colistin in the treatment of pneumonia in critically ill patients. Of two recent studies evaluating combined aerosolized colistin and intravenous colistin in patients with ventilator-associated pneumonia [7,8], only one study showed significant benefit [8].

It therefore remains unclear whether adding aerosolized colistin to intravenous colistin improves outcomes in critically ill patients with pneumonia. In this retrospective study we examine the efficacy of adjunctive aerosolized colistin for the treatment of pneumonia caused by MDR-GN organisms in critically ill patients.

## Methods

### Study location and patient population

This study was Institutional Review Board-approved and conducted at three tertiary-care academic medical centers: The Ohio State University Wexner Medical Center (OSUWMC) in Columbus, Ohio; Mayo Clinic (Mayo) in Rochester, Minnesota; and Barnes-Jewish Hospital (BJH) in St. Louis, Missouri. This study was approved by the Ohio State University Biomedical Sciences Institutional Review Board, the Mayo Clinic Institutional Review Board and the Washington University School of Medicine Human Studies Committee. Patients >18 years of age admitted to an ICU between July 2007 and July 2009 were eligible for evaluation. Included patients received intravenous colistin for at least 48 hours with or without aerosolized colistin for the management of MDR-GN pneumonia. Based on the Center for Disease Prevention and Control criteria, pneumonia was defined as temperature < 35.5°C or > 38°C and leukocytosis >12,000 cells/mm^3^ or leukopenia <4,000 cells/mm^3^ with signs and symptoms consistent with pneumonia, in combination with a positive respiratory culture obtained by bronchoalveolar lavage (BAL), sputum or tracheal aspirate [9]. Patients with concomitant infections were included as long as they had appropriate antimicrobial coverage for the infection. Patients were excluded if they were incarcerated or pregnant, or had a history of cystic fibrosis or lung transplantation. If a patient met inclusion criteria on multiple occasions, only the first episode was evaluated.

### Study design and data collection

This was a retrospective, three-center, cohort analysis comparing critically ill patients with nosocomial pneumonia who received intravenous colistin (IV) alone or in combination with adjunctive aerosolized colistin (IV/AER). The primary outcome was the incidence of clinical cure. Secondary endpoints included microbiologic cure, duration of mechanical ventilation, length of stay, all-cause hospital mortality and MDR-GN pneumonia attributable mortality. A post-hoc analysis was performed comparing IV and IV/AER for the subgroup of patients diagnosed by BAL. For the purpose of this study, colistin will be referred to as milligrams (mg) in colistin base. It is standard practice at each institution for the ICU pharmacist to review the dosing of colistin prior to dispensing. Although optimal dosing for colistin is unknown, a standardized hospital guideline was used at each institution to ensure adequate dosing and frequency based on body weight, renal function, and volume status of the patient (Table 1). The decision to add adjunctive aerosolized therapy to IV colistin was based on provider preference. All institutions utilize nebulizers that generate an optimal mean mass aerodynamic diameter of the aerosol particles of 1–5 μm for delivery of aerosolized colistin [10]. Two centers, OSUWMC and BJH, employed a jet nebulizer for administration of aerosolized colistin, while a vibrating mesh technology was used at Mayo.

**Table 1:** Institutional guidelines for intravenous and aerosolized colistin dosing

	**The Ohio State University Wexner Medical Center**	**Mayo clinic**	**Barnes-Jewish hospital**
Intravenous colistin			
Loading dose	None	None	None
Maintenance dose	CrCl>70 ml/min: 2.5 mg/kg every 12 hours	CrCl >80 ml/min: 5 mg/kg/day divided in two doses	CrCl>80 ml/min: 2.5 mg/kg every 12 hours
	CrCl 30-70 ml/min: 1.5 mg/kg every 24 hours	CrCl 50-80 mL/min: 2.5-3.8mg/kg/day divided in two doses	CrCl 40-80 ml/min: 1.25-1.9 mg/kg every 12 hours
	CrCl<30 ml/min: 1.5 mg every 48 hours	CrCl 10-49 ml/min: 2.5 mg/kg every 24 hours	CrCl 25-40 ml/min: 1.25 mg/kg every 24 hours
	IHD: 1.5 mg/kg every 48 hours after dialysis	CrCl <10 ml/min: 1.5 mg/kg every 24 hours	CrCl10-25 ml/min: 1.5 mg/kg every 36 hours
	CRRT: 2.5 mg/kg every 24 hours	IHD: 1.5 mg/kg every 24 hours or 2-3mg/kg after dialysis only on dialysis days	CrCl<10 ml/min: 1.5 mg every 48 hours
		CRRT: 2.5 mg/kg every 12-24 hours	IHD: 1.5 mg/kg three times weekly after dialysis only on dialysis days
			CRRT: 2.5 mg/kg every 48 hours
Aerosolized colistin			
Dosing	75 mg every 12 hours	75 mg every 12 hours	150 mg every 12 hours
Nebulizer	Jet	Vibrating mesh	Jet

All BALs were conducted via bronchoscope with the exception of those collected in the surgical ICU at OSUWMC where all were performed blind. Non-BAL cultures may represent a sputum sample or tracheal aspirate. Due to the labeling of both sputum and tracheal aspirates as sputum specimens, these samples are grouped together as sputum/tracheal aspirate. Quantitative methods for culture assessment were used at all centers. The threshold for diagnosis of infection was an organism growing at greater than 10,000 colony forming units per milliliter.

The following baseline characteristics were collected from the medical records of eligible patients: age, gender, co-morbidities, Acute Physiology and Chronic Health Evaluation (APACHE) II score at ICU admission, and healthcare exposure and antibiotic use in the previous 90 days. Patients were considered immunosuppressed if they were receiving an immunosuppressant (i.e. chemotherapy, calcineurin inhibitor, sirolimus) or corticosteroids equal to or greater than prednisone 20 mg per day. Any microbiologic history of a previous MDR organism was collected for all patients, including colonization and infection. In addition, the following variables were recorded: microbiologic culture data including organisms and antibiotic susceptibilities, time to appropriate antimicrobial coverage, concomitant antimicrobials, colistin dosing and duration, and assessment of appropriate antimicrobial coverage of other organisms.

### Definitions

Clinical cure was defined as resolution of initial signs and symptoms of infection including normalization of white blood cell count and temperature by the end of colistin therapy. Microbiological cure was defined as eradication of the MDR pathogen on subsequent respiratory cultures. Pathogens were considered MDR if they were resistant to at least one agent in three or more antimicrobial categories to which the organism would typically be susceptible. Initial total daily doses of intravenous colistin were categorized as adequate if they were at least 80% of the recommended dose based on institutional guidelines. Additional coverage was defined as the addition of an antibiotic to intravenous colistin to which the MDR-GN organism’s sensitivity was reported as either intermediate or susceptible.

### Statistical analysis

Dichotomous data are expressed as frequency distributions and were compared using the Fisher’s exact test. Normally distributed continuous data are expressed as mean ± standard deviation and were compared using the student’s t-test. For non-parametric continuous data, values are presented as median [interquartile range] and were compared using the Mann–Whitney U test. All tests were two-tailed and a p-value of less than 0.05 indicates statistical significance. All analyses were conducted using SPSS 17.0 (SPSS, Inc; Chicago, IL).

## Results

Ninety five patients with MDR-GN pneumonia were studied: 51 patients received IV and 44 patients received IV/AER colistin. There were 69 patients included from OSUWMC, 22 patients from Mayo and 4 patients from BJH. There were no statistically significant differences in baseline characteristics between the two groups, although more patients in the IV group had a history of an extended-spectrum beta lactamase (ESBL) organism (Table 2).

**Table 2:** Baseline characteristics

**Characteristic**^***a***^	**Intravenous colistin only (n = 51)**	**Intravenous + aerosolized colistin (n = 44)**	**p- value**
Age (years)	57.3 ± 15.6	60.9 ± 15.3	0.255
Male	33 (64.7)	22 (50)	0.148
ICU			0.279
Medical	30 (58.8)	21 (47.7)	
Surgical	21 (41.2)	23 (52.3)	
Co-morbidities			
Hypertension	30 (58.8)	33 (75.0)	0.096
Diabetes	17 (33.3)	19 (43.2)	0.324
CKD	10 (19.6)	7 (15.9)	0.639
ESRD	6 (11.8)	2 (4.5)	0.279
Solid organ transplant	3 (5.9)	0	0.246
BMT	1 (2.0)	1 (2.3)	>0.99
Hematologic malignancy	5 (9.8)	4 (9.1)	>0.99
Solid malignancy	12 (23.5)	5 (11.4)	0.123
Chronic immunosuppressive therapy	21 (41.2)	13 (29.5)	0.238
Microbiologic history			
*Acinetobacter spp.*	5 (9.8)	5 (11.4)	>0.99
*Pseudomonas spp.*	21 (41.2)	12 (27.3)	0.156
VRE	15 (29.4)	12 (27.3)	>0.99
MRSA	19 (37.3)	18 (40.9)	0.716
ESBL	10 (19.6)	1 (2.3)	0.008
Hospital admission within last 90 days	25 (49.0)	23 (52.3)	0.752
Broad spectrum antibiotics within last 90 days	33 (64.7)	23 (52.3)	0.219
Transfer from long-term care facility	18 (35.3)	14 (31.8)	0.721
Transfer from outside hospital	16 (31.4)	10 (22.7)	0.346
APACHE II score	24 ± 6.9	22.4 ± 7.1	0.266
Serum creatinine at colistin initiation (mg/dl)	1.3 ± 1.1	1.4 ± 0.9	0.808
Mechanical ventilation	49 (96.1)	42 (95.5)	>0.999

^*a*^Values are expressed as number (%) and mean ± SD unless otherwise stated.

*Abbreviations:* ICU = intensive care unit, CKD = chronic kidney disease, ESRD = end-stage renal disease, BMT = stem cell transplant, VRE = vancomycin-resistant *Enteroccocus,* MRSA = methicillin-resistant *Staphylococcus aureus,* ESBL = extended-spectrum beta-lactamase, APACHE = Acute Physiology and Chronic Health Evaluation.

The most common MDR-GN pathogens identified included *A. baumannii* (64.2%) and *P. aeruginosa* (55.8%) (Table 3). Twenty-five patients had more than one MDR-GN organism cultured from the same respiratory sample. There were no colistin-resistant strains isolated from either group. Patients infected with *A. baumannii* were more likely to receive IV/AER (82 vs. 49%, p = 0.001), while patients infected with *P. aeruginosa* were more likely to receive only IV (68.6 vs. 40.9%, p = 0.007). Some patients also had extrapulmonary MDR-GN infections including blood (n = 24), urine (n = 15), skin/soft-tissue (n = 8), and other (n = 6). There were no differences in distribution of co-infection sites between the two groups.

**Table 3:** Microbiologic data and treatment

**Variable**^***a***^	**Intravenous colistin only (n = 51)**	**Intravenous + aerosolized colistin (n = 44)**	**p-value**
Respiratory source, n (%)			
Bronchoalveolar lavage	32 (62.7)	35 (79.5)	0.113
Sputum/tracheal aspirate	19 (37.3)	9 (20.5)	0.113
Respiratory isolates^*b*^			
*Acinetobacter spp.*	25 (49.0)	36 (81.8)	0.001
*Pseudomonas spp.*	35 (68.6)	18 (40.9)	0.007
ESBL *Klebsiella spp.*	9 (17.6)	2 (4.5)	0.047
Other site of infection			
Blood	10 (19.6)	14 (31.8)	0.172
Urine	9 (17.6)	6 (13.6)	0.593
SSTI	4 (7.8)	4 (9.1)	>0.99
Other	5 (9.8)	1 (2.3)	0.211
Additional coverage with colistin	33 (64.7)	19 (43.2)	0.036
Tigecycline	7 (21.2)	9 (42.9)	0.089
Aminoglycoside	8 (24.2)	3 (14.3)	0.497
Carbapenem	13 (39.4)	5 (23.8)	0.236
Piperacillin/tazobactam	3 (9.1)	0	0.274
Ampicillin/sulbactam	1 (3.0)	2 (9.5)	0.553
Cefepime	5 (15.2)	1 (4.8)	0.386
Ciprofloxacin	1 (3.0)	1 (4.8)	>0.99
Intravenous colistin dosing			
Total daily dose IBW (mg/kg)	3.7 ± 2.1	4 ± 2.3	0.596
Total daily dose TBW (mg/kg)	2.5 ± 1.5	2.6 ± 1.4	0.654
Time to appropriate therapy (days), median [IQR]	4 [3–6.25]	4 [3–5.75]	0.625
Duration of intravenous colistin (days)	11.2 ± 7.7	12.2 ±7.2	0.529
Total intravenous dose (mg), median [IQR]	2100 [700–4200]	2002.5 [1080–4200]	0.407
Adequate initial intravenous dosing	38 (74.5)	33 (77.3)	0.754
Aerosolized colistin dosing			
Total daily dose (mg), median [IQR]		75 [75–75]	NA
Duration (days), median [IQR]		11.0 [7–16.25]	NA

^*a*^Values are expressed as number (%) and mean ± SD unless otherwise stated.

^*b*^25 patients had more than one MDR organism cultured from the same respiratory sample.

*Abbreviations:* ESBL = extended-spectrum beta-lactamase, SSTI = skin and soft tissue infection,IBW = ideal body weight, TBW = total body weight, IQR = interquartile range.

Time to appropriate therapy for the MDR organism was similar between IV and IV/AER patients (4 [3–6.25] vs. 4 [3–5.75] days, p = 0.625). The mean initial intravenous colistin total daily dose was similar between the IV and IV/AER groups with no difference in adequacy of dosing of intravenous colistin between the IV and IV/AER patients (Table 3). Patients in the IV group were more likely to receive additional intravenous antibiotics (64.7 vs. 43.2%, p = 0.036), although, there were no differences between the groups when the individual antibiotics used as additional coverage were analyzed (Table 3).

**Figure 1:**
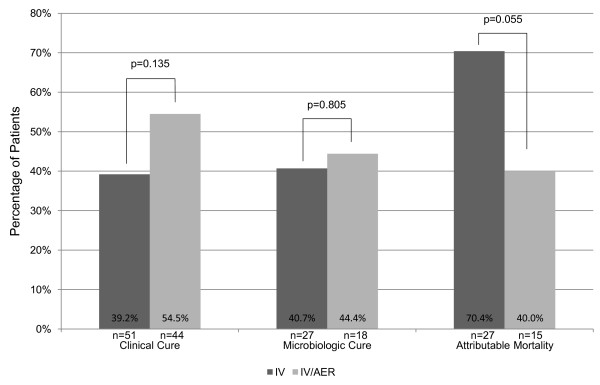
Outcomes of intravenous colistin compared to intravenous plus aerosolized colistin for multi-drug resistant gram-negative pneumonia.

Twenty patients (39.2%) receiving IV and 24 patients (54.5%) receiving IV/AER achieved clinical cure (p = 0.135) (Figure 1). A total of 45 patients were evaluable for microbiologic cure. Eleven IV patients (40.7%) and 8 IV/AER patients (44.4%) attained microbiologic cure (p = 0.805). In patients receiving IV compared to IV/AER, the median duration of mechanical ventilation (21.51 [8.36-40.5] vs. 21.65 [11.75-35] days, p = 0.799), ICU length of stay (23 [9–51] vs. 24.5 [15.25-49] days, p = 0.657) and hospital length of stay (40 [17–61.46] vs. 33 [20.99-54.75] days, p = 0.734) were not significantly different. The difference in hospital mortality rates between the IV and the IV/AER groups were not statistically significantly different (52.9 vs. 36.4%, p = 0.106). Pneumonia attributable mortality was also higher among patients that received IV compared to IV/AER, although this failed to reach statistical significance (70.4 vs. 40%, p = 0.055).

**Figure 2:**
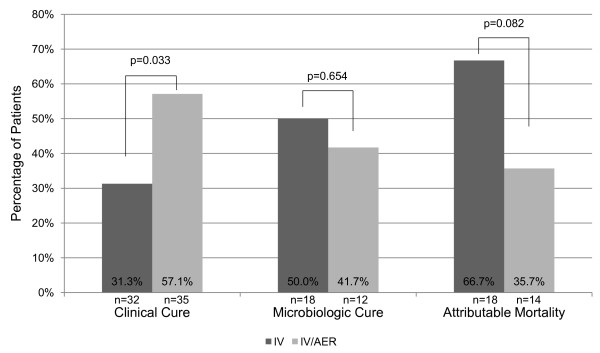
Subgroup with high quality respiratory cultures: outcomes of intravenous colistin compared to intravenous plus aerosolized colistin for multi-drug resistant gram-negative pneumonia.

In the subgroup of patients diagnosed by high quality culture (BAL), patients who received IV had a significantly lower incidence of clinical care compared to those who received IV/AER (31.3 vs. 57.1%, p = 0.033) (Figure 2). Pneumonia attributable mortality among the IV group was not statistically higher compared to the IV/AER group (66.7 vs. 35.7%, p = 0.082).

## Discussion

Few prospective studies have evaluated the role of aerosolized colistin specifically in critically ill patients with MDR-GN pneumonia, likely due to the low incidence of these infections in a single-center setting. In the primary analysis, the current study failed to demonstrate a statistically significant benefit to IV/AER over IV. However, there was trend towards a higher clinical cure rate and decreased mortality, and the subgroup analysis of high quality specimens showed a significantly higher rate of clinical cure with IV/AER.

Given the increased morbidity and mortality associated with MDR-GN infections in critically ill patients, it is important to determine if aerosolized colistin confers added benefit to systemic infusion [7]. In theory, direct delivery of an antibiotic to the site of infection should be beneficial and might limit systemic side effects. Aerosolized colistin is well tolerated with the exception of rare cases of bronchoconstriction [11]. Although promising, the early studies of aerosolized colistin both as combination and monotherapy, did not include a comparator group [12-14]. To date, there are only two published studies comparing intravenous colistin alone to intravenous plus aerosolized colistin [7,8]. Korbila et al. retrospectively observed 121 patients with ventilator-associated pneumonia caused by MDR-GN pathogens and found a significantly higher clinical cure rate (79.5 vs. 60.5%, p = 0.025) in patients that received aerosolized colistin in conjunction with systemic therapy, however failed to demonstrate a mortality difference between the two groups (44.2% vs. 39.7%, p = 0.63) [8]. Another retrospective case–control study evaluated 86 age and APACHE II matched patients and found that 54% of those receiving IV/AER colistin achieved clinical cure compared to 32.5% in the IV colistin group (p = 0.05) [7]. In addition, the authors found a trend towards reduced all-cause mortality when patients received IV/AER colistin therapy (42% vs. 23%, p = 0.066). Data from our study corroborates findings from these earlier reports, with a trend towards a higher rate of clinical cure in patients receiving IV/AER colistin and a significantly higher rate when only evaluating patients with a high quality culture. Neither the two previous studies, nor the current results, definitively demonstrate mortality difference between IV and IV/AER colistin therapy, although in all the studies clinical cure was the primary outcome. The lack of statistical significance for clinical cure in the study by Kofteridis et al. as well as our study may be due to several factors, including relatively small sample sizes and selection bias (i.e., patients may have received adjunctive aerosolized colistin due to a higher severity of illness). In addition, our study included a considerable number of immunosuppressed patients. This patient population is generally excluded in other studies and may have negatively impacted the clinical cure rate.

Despite evidence suggesting higher clinical cure rates and survival benefit with adjunctive aerosolized colistin, no studies have found improvement in microbiologic outcomes [7,8]. Our study suggested no difference in microbiologic cure rates when comparing IV to IV/AER colistin therapy. However, retrospective evaluation of microbiologic cure is not optimal as a determinant of therapeutic efficacy due to the bias inherent to repeat respiratory cultures. It is currently not routine practice to perform follow-up respiratory cultures at the centers included in this study. Patients unresponsive to ongoing treatment may be more likely to undergo subsequent cultures, while patients with clinical cure are less likely to have follow-up cultures. An additional factor limiting the utility of microbiologic cure as an outcome measure is the inherent challenge of differentiating bacterial pneumonia from colonization following the initial infection [15]. When evaluating persistence of respiratory tract colonization in critically ill, mechanically ventilated patients, Visscher et al. showed high rates of persistent colonization with both *Acinetobacter* spp (56%) and *P. aeruginosa* (85%). In addition, in these two pathogens only, antibiotic therapy with adequate coverage had no impact on rates of persistent colonization [16]. The near-universal use of acid-lowering agents in stress ulcer prophylaxis has been associated with significant rise of GN colonization of the lower airway and the incidence of GN bacterial colonization in critically ill, ventilated patients is prevalent despite susceptibility to antimicrobial therapy [17,18]. These findings, when combined with the overall results of this study, highlight the controversy regarding microbiologic versus clinical cure as the more relevant endpoint in patients with MDR-GN pneumonia.

Utilization of other antibiotics in addition to colistin between the two groups could significantly influence outcomes. The addition of another antibiotic to colistin has been previously studied in the treatment of nosocomial pneumonia caused by MDR gram-negative organisms [19]. In our study, there was a higher proportion of patients infected with *P. aeruginosa* in the IV group, whereas more patients receiving IV/AER colistin had *A. baumannii* infections. When evaluating antibiotics used for additional coverage individually, there was no difference between the two groups, but overall there was more additional antibiotic coverage utilized in the IV group. The most common antibiotics for additional coverage were carbapenems, followed by tigecycline and aminoglycosides. There was a trend towards increased tigecycline use in the IV/AER group, which may correlate to the increased incidence of *A. baumannii* in this group*.* In addition, there was increased use of carbapanems and aminoglycosides in the IV only group, correlating to the larger proportion of *P. aeruginosa* isolated in this group. Clinical studies have not shown significant benefit of additional intravenous coverage over colistin monotherapy [19]. However, in vitro studies have found that addition of a second antibiotic to colistin may be beneficial for infections caused by *P. aeruginosa.* In a study by Rynn et al., the addition of an antipseudomonal agent to colistin produced greater killing of *P. aeruginosa* than either agent alone [20]. Synergistic effects have also been demonstrated between meropenem and colistin in *A. baumannii* strains [21]. Unfortunately, because of the different distribution of *P. aeruginosa and A. baumannii* between the groups it is difficult to interpret the role of additional coverage in the outcomes of our study.

While the clinical outcomes evaluated (clinical cure, mortality and microbiologic cure) were consistent with previous literature reports, our study has several characteristics that may increase applicability of results. The inclusion of patients from multiple centers and all ICU subtypes increases the likelihood the results are relevant to many critically ill patient populations. In addition to assessing dosing and duration of systemic colistin, we standardized systemic colistin dosing using hospital based guidelines. Moreover, similar total numbers of *Acinetobacter* and *Pseudomonas* were included in this evaluation. While previous studies also included all MDR-GN organisms, the proportion *Pseudomonas spp* infections was small, thus limiting applicability of the results to this pathogen.

Despite these strengths, our study has several limitations. Although we used a multi-center design, our sample size lacked power to detect differences between groups. In addition, differences in *A. baumannii and P. aeruginosa* distribution make it difficult to determine the impact of additional intravenous coverage on outcomes. Additionally, we included sputum and tracheal aspirate specimens in the diagnostic criteria for pneumonia which have inferior sensitivity and specificity for diagnosis of pneumonia when compared to BAL. However, a subgroup analysis was performed to highlight the differences between IV and IV/AER in patients with high quality respiratory specimens.

## Conclusion

Our study demonstrated an increased rate of clinical cure and trend towards improved pneumonia attributable mortality for patients diagnosed with BAL who received combination aerosolized-intravenous colistin therapy when compared to intravenous colistin alone for MDR-GN pneumonia. These findings support the use of aerosolized colistin as an adjunct to intravenous colistin and also highlight the need for additional, larger prospective clinical trials to confirm the benefit of aerosolized colistin as adjunctive therapy in the treatment of nosocomial MDR-GN pneumonia in critically ill patients.

## Abbreviations

APACHE II: Acute Physiology and Chronic Health Evaluation II; BAL: Bronchoalveolar lavage; BMT: Stem cell transplant; CF: Cystic fibrosis; CKD: Chronic kidney disease; ESBL: Extended-spectrum beta-lactamase; ESRD: End-stage renal disease; IBW: Ideal body weight; ICU: Intensive care unit; IV: Intravenous colistin; IV/AER: Intravenous plus aerosolized colistin; MDR: Multi-drug resistant; MDR-GN: Multi-drug resistant gram negative; MRSA: Methicillin-resistant *Staphylococcus aureus*; SSTI: Skin and soft tissue infection; TBW: Total body weight; VRE: Vancomycin-resistant *Enteroccocus*.

## Competing interests

The authors declare that they have no competing interests.

## Authors’ contributions

ND participated in the study design, was the primary data collector, oversaw database management, contributed to results interpretation, and drafted the manuscript. CC contributed to study design, results interpretation, and manuscript revisions for intellectual content. KM participated in study design, assisted with data collection, results interpretation, and provided manuscript revisions for intellectual content. SS contributed specifically to the study design, statistical analysis and results interpretation, and provided revisions for intellectual content for the manuscript in its entirety. EF, HP, GS and HA conducted data acquisition for their respective institutions and were involved in manuscript revisions for intellectual content. CM conceived of the study concept, contributed to study design, provided study supervision, statistical analysis, results interpretation, and manuscript revisions for intellectual content. All authors read and approved the final manuscript.

## Pre-publication history

The pre-publication history for this paper can be accessed here:

http://www.biomedcentral.com/1471-2253/13/45/prepub
